# Dose-response relationship between lower serum magnesium level and higher prevalence of knee chondrocalcinosis

**DOI:** 10.1186/s13075-017-1450-6

**Published:** 2017-10-24

**Authors:** Chao Zeng, Jie Wei, Robert Terkeltaub, Tuo Yang, Hyon K. Choi, Yi-lun Wang, Dong-xing Xie, David J. Hunter, Yuqing Zhang, Hui Li, Yang Cui, Liang-jun Li, Guang-hua Lei

**Affiliations:** 1 Department of Orthopaedics, Xiangya Hospital, Central South University, #87 Xiangya Road, Changsha, Hunan Province 410008 China; 2 Health Management Center, Xiangya Hospital, Central South University, Changsha, Hunan Province 410008 China; 3 VA San Diego Medical Center, San Diego, CA 92161 USA; 4 Department of Medicine, UCSD, San Diego, CA 92161 USA; 5 Division of Rheumatology, Allergy, and Immunology, Department of Medicine, Massachusetts General Hospital, Boston, MA 02114 USA; 6 Rheumatology Department, Royal North Shore Hospital and Institute of Bone and Joint Research, Kolling Institute, University of Sydney, Sydney, NSW 2065 Australia; 7 Department of Epidemiology and Health Statistics, Xiangya School of Public Health, Central South University, Changsha, Hunan Province 410008 China; 8 International Medical Center, Xiangya Hospital, Central South University, Changsha, Hunan Province 410008 China; 9 Department of Orthopaedics, Changsha Central Hospital, Changsha, Hunan 410000 China

**Keywords:** Chondrocalcinosis, Magnesium, Knee

## Abstract

**Background:**

The aim was to assess serum magnesium levels in relation to prevalence of knee chondrocalcinosis in two population-based Chinese studies.

**Methods:**

Data included in this analysis consisted of two population-based cross-sectional studies, i.e., the Xiangya Hospital Health Management Center Study and the Xiangya Osteoarthritis (XO) Study I. A bilateral knee anteroposterior radiograph was obtained from each subject. Radiographic knee chondrocalcinosis was present if there was definite linear cartilage calcification. Serum magnesium concentration was measured using the chemiluminescence method. We examined the relation of serum magnesium levels to prevalence of knee chondrocalcinosis using generalized estimating equations.

**Results:**

The prevalence of knee chondrocalcinosis was 1.4% in the Xiangya Hospital Health Management Center Study (n = 12,631). Compared with the lowest tertile, the age, sex and body mass index (BMI)-adjusted odds ratios (ORs) of chondrocalcinosis were 0.59 (95% CI 0.40–0.87) and 0.49 (95% CI 0.33–0.72) in the second and the third tertiles of serum magnesium, respectively (*P* for trend <0.001). The prevalence of knee chondrocalcinosis in the XO Study I (n = 1316) was 4.1%. The age, sex and BMI-adjusted ORs of chondrocalcinosis were 0.67 (95% CI 0.34–1.30) in the second and 0.45 (95% CI 0.21–0.94) in the third tertile of serum magnesium when compared with the lowest tertile (*P* for trend = 0.030). Similar results were observed in men and women in both studies. Adjusting for additional potential confounders did not change the results materially.

**Conclusions:**

Subjects with lower levels of serum magnesium, even within the normal range, had higher prevalence of knee chondrocalcinosis in a dose-response relationship manner, suggesting that magnesium may have a preventive or therapeutic potential for knee chondrocalcinosis.

**Electronic supplementary material:**

The online version of this article (doi:10.1186/s13075-017-1450-6) contains supplementary material, which is available to authorized users.

## Background

Calcium pyrophosphate deposition (CPPD) in articular fibrocartilage and hyaline cartilage, termed chondrocalcinosis, has been considered to be associated with both aging and osteoarthritis [1]. CPPD, together with basic calcium phosphate (BCP), are the two main components of calcium-containing crystals in cartilage in osteoarthritis [2, 3]. While most subjects with chondrocalcinosis have no clinical symptoms (asymptomatic CPPD) [4], CPPD may promote articular degeneration (osteoarthritis with CPPD) and traffic of the crystals from articular cartilage into the joint space; and thus can stimulate acute episodic crystal arthritis and can also lead to chronic inflammatory arthritis.

To date, only a few risk factors have been identified for the occurrence of chondrocalcinosis. Studies have shown that prevalence of chondrocalcinosis varied in different ethnic groups. For example, prevalence of chondrocalcinosis was much lower in Beijing Osteoarthritis Study participants (1.8% in men, 2.7% in women) than that in white subjects among the predominantly white participants in the Framingham Study (6.2% in men, 7.7% in women) [5]. Several studies also found that knee chondrocalcinosis is associated with low levels of serum magnesium [6–11]. For instance, patients with hypomagnesemia (e.g., poor parenteral nutrition, short bowel syndrome, Gitelman syndrome or familial heredity) had a much higher prevalence of chondrocalcinosis (up to 23.1%) [6–11]. However, these findings were often based on subjects with extremely low serum magnesium, and it remains unclear whether more modest variations of magnesium levels observed in the general population are associated with the prevalence of chondrocalcinosis. Such potential associations and their quantification would have an implication in public health and clinical practices.

To fill in this knowledge gap, we used data collected from two large population-based studies (i.e., Xiangya Hospital Health Management Center Study and Xiangya Osteoarthritis (XO) Study I) and examined the relation of serum magnesium levels with the prevalence of knee chondrocalcinosis and to determine the shape of the dose-response relationship between serum magnesium levels and the prevalence of knee chondrocalcinosis.

## Methods

### Study population

#### Xiangya Hospital Health Management Center Study

Subjects included in this study were residents living in Hunan, China, who were undergoing routine health examination at Xiangya Hospital, Central South University, China. The study design has been published elsewhere [12–14]. In brief, routine health checkup included anthropometric (e.g., height, weight, etc.), basic clinical examination (e.g., blood pressure, heart rate, etc.), and biochemical (e.g., blood routine examination, hepatic function, renal function, trace elements test, etc.) and imaging tests (e.g., chest radiography, weight-bearing bilateral anteroposterior knee radiography, etc.). Subjects included in the current analysis were those who: (1) had health checkup between October 2013 and December 2015; (2) were age ≥40 years; (3) underwent a serum magnesium test; and (4) had bilateral weight-bearing anteroposterior radiographs. All interviewers, clinical examiners and x-ray technicians were trained by the principal investigators (CZ and GL) before the study began.

Of 14,715 participants who underwent routine health examination, 2081 (14.1%) were excluded from the analysis for the following reasons: (1) age <40 years old (n = 1408); (2) low quality of weight-bearing anteroposterior knee radiographs (n = 63); (3) the Kellgren-Lawrence (K-L) [15] grades of both knees were 4 (n = 8); and (4) repeated health examinations in the same person during the study period (n = 604). Finally, 12,631 participants were included in the analysis.

#### Xiangya Osteoarthritis Study I (XO Study I)

Subjects included in this study were a randomly selected sample of residents, age ≥50 years, from eight rural mountainous communities of Longshan County [16], Hunan Province, for a study of osteoarthritis. All of the villages in the selected communities were listed in a random order. Beginning with the first village in the first community, all residents age ≥50 years were invited to participate in our study. The village-to-village recruitment continued until the number of subjects in that community met the predetermined quota according to the Sixth National Census Data of Longshan County (2010). Subjects were recruited at the site near their home and were transported to the hospital for interview and clinical examination between November 2015 and January 2016. Trained health professional interviewers administered a standardized questionnaire that focused on sociodemographic factors, lifestyle habits, joint symptoms, joint functions, quality of life, dietary intake, medication use and other potential risk factors for osteoarthritis. Clinical examination, laboratory testing and radiography were also conducted at the time of interview. All interviewers, clinical examiners and x-ray technicians were trained under the supervision of the study principal investigators (CZ and GL).

Of 1739 age-eligible (i.e., age ≥50 years) residents randomly selected in Longshan County, 270 (15.5%) subjects declined to participate the study. There was no significant difference in sex and age distribution between subjects who consented to participate and who declined (*P* = 0.42 and 0.47, respectively). Among 1469 participants in the XO Study I, 153 were excluded from the analysis because of: (1) history of lower limb surgery (n = 37); (2) history of pelvic surgery (n = 1); (3) lower extremity disability due to cerebrovascular diseases (n = 2) or spinal diseases (n = 2); (4) rheumatoid arthritis (n = 61); (5) unavailability of knee radiographs (n = 3); (6) low quality of weight-bearing anteroposterior radiographs of the knees (n = 1); (7) K-L grades of 4 (n = 5) in both knees; and (8) unavailability of a serum magnesium test (n = 41). Finally, 1316 participants were included in the analysis.

### Blood biochemical analysis

All blood samples were drawn after a 12-hour overnight fast and were stored at 4 °C until analysis. The serum magnesium concentration was measured using the chemiluminescence method by Beckman Coulter AU 5800 (Beckman Coulter Inc., Brea, CA, USA). The inter-assay and intra-assay coefficients of variation were tested by low concentrations (0.60 mmol/L of serum magnesium) and high concentrations (1.00 mmol/L of serum magnesium) of standard human samples. The intra-assay coefficients of variation were 1.86% (0.60 mmol/L) and 1.65% (1.00 mmol/L) for serum magnesium, and the inter-assay coefficients of variation were 1.87% (0.60 mmol/L) and 1.70% (1.00 mmol/L) for serum magnesium. Measuring methods and reliability data of potential confounders (e.g., serum parathyroid hormone, iron, ferritin, total iron binding capacity, calcium, copper, zinc, phosphorus and vitamin D) are shown in Additional file 1.

### Assessment of radiographic knee chondrocalcinosis

All radiographs were assessed by two orthopedic surgeons (TY and YLW) who were blinded to subjects’ clinical symptoms and biochemical test results. Radiographic chondrocalcinosis was defined as present when there was evidence of definite linear cartilage calcification in the knee [17]. Specifically, prior to starting the assessment, these two orthopedists re-read 200 radiographs from the Osteoarthritis Initiative (OAI) to calibrate their reading. Formal readings of batches of randomly selected radiographs did not start until the readers reached a high level of agreement with previous readings from OAI (the specific cutoff of simple kappa for inter-rater reliability was 0.70). During the formal reading, one batch of knee radiographs (100 radiographs) consisting of 10 previously read radiographs selected at random and 90 unread radiographs were mingled and read. For each batch, 90 unread radiographs were used to test inter-rater reliability and 10 previously read radiographs were used to test intra-rater reliability, respectively. Two readers both assessed all radiographs and inconsistencies were resolved through discussion. The kappa value for reliability readings of radiographs from Xiangya Hospital Health Management Center Study was 0.72 (95% CI 0.67 − 0.77) for inter-rater reliability and 0.76 (95% CI 0.67–0.85) for intra-rater reliability. The kappa value for reliability readings of radiographs from XO Study I was 0.75 (95% CI 0.68–0.83) for inter-rater reliability and 0.80 (95% CI 0.65–0.94) for intra-rater reliability.

### Statistical analysis

Continuous data were expressed as the mean ± standard deviation, and categorical data were expressed as proportion (percentage). The serum magnesium concentration was classified into three categories based on the tertiles distribution in each study population (i.e., ≤ 0.86, 0.87–0.91 and ≥ 0.92 mmol/L in the Xiangya Hospital Health Management Center Study and ≤ 0.89, 0.90–0.95 and ≥ 0.96 mmol/L in the XO Study I). We calculated the sex-specific prevalence of chondrocalcinosis for each category of serum magnesium. We examined the association of serum magnesium categories and prevalence of chondrocalcinosis using generalized estimating equations (GEE) [18], adjusting for the potential confounders (knee specific analysis). Odds ratios (OR) and related 95% confidence intervals (95% CI) of chondrocalcinosis among different categories of serum magnesium were calculated using the PROC GENMOD procedure in SAS with binomial distribution and logit links, and the lowest tertile of serum magnesium was considered as the reference. We also calculated the OR and the related 95% CI by using the category ≤0.70 mmol/L (dividing the lowest category of serum magnesium into two categories: ≤0.70 mmol/L and 0.71–0.86 in the Xiangya Hospital Health Management Center Study, and the category ≤0.70 mmol/L and 0.71–0.89 in the XO Study I) as the reference group. Specifically, in the Xiangya Hospital Health Management Center Study we first adjusted for age (40–49, 50–59, 60–69, ≥70 years), body mass index (BMI) (<25, ≥25 kg/m^2^) and sex (male, female). Then we added each of the following covariates separately: serum iron (tertiles), serum ferritin (tertiles), serum calcium, serum zinc (tertiles), serum copper (tertiles), serum phosphorus (tertiles), education (high school or above, lower than high school) and occupation (manual labor, non-manual labor) in the regression model adjusted for age, sex and BMI. In the XO Study I, we first adjusted for age (50–59, 60–69, ≥70 years), BMI (<25, ≥25 kg/m^2^) and sex (male, female), and then added each of the following covariates separately in the regression models. These covariates included knee injury, serum parathyroid (tertiles), serum iron (tertiles), serum total iron binding capacity (tertiles), serum unsaturated iron binding capacity (tertiles), serum calcium (tertiles), serum 25(OH)D (tertiles), serum zinc (tertiles), serum copper (tertiles), serum phosphorus (tertiles), education (educated, non-educated) and occupation (farmer, non-farmer).

In addition, we performed sex-specific analysis to examine the levels of serum magnesium and prevalence of chondrocalcinosis. The dose-response relationship between levels of serum magnesium and the prevalence of knee chondrocalcinosis was evaluated by restricted cubic splines regression with two knots defined by the tertile distribution of serum magnesium [19, 20]. We also used the value 0.7 mmol/L as an additional knot, to evaluate the dose-response association between serum magnesium and the prevalence of knee chondrocalcinosis.

## Results

### Xiangya Hospital Health Management Center Study

Of the remaining 12631 participants from the initially included 14,715 participants, 43.0% (n = 5428) were women, the average age was 52.3 ± 8.0 years, and the mean BMI was 24.5 kg/m^2^ (SD = 3.3 kg/m^2^). The prevalence of knee chondrocalcinosis was 1.3% in men and 1.5% in women (Table 1).

**Table 1:** Basic characteristics of the population in the Xiangya Hospital Health Management Center Study (n = 12,631)

	Tertiles of serum Mg (mmol/L)		
	1 (≤0.86)	2 (0.87–0.91)	3 (≥0.92)
Number	4633	3720	4278
Median Mg level (mmol/L)	0.83	0.89	0.95
Sex (female, %)	45.3	42.5	40.9
Age (years)	52.1 ± 8.1	52.4 ± 8.1	52.5 ± 7.9
40–49 (%)	43.5	41.1	39.5
50–59 (%)	37.5	39.5	40.4
60–69 (%)	15.2	15.9	17.2
≥70 (%)	3.8	3.5	2.9
BMI (kg/m^2^)	24.5 ± 3.4	24.4 ± 3.3	24.4 ± 3.2
<25 (%)	57.6	59.2	58.6
≥25 (%)	42.4	40.8	41.4
Serum iron (μmol/L)	18.1 ± 6.5	18.4 ± 6.3	18.9 ± 6.5
Serum ferritin (μg/L)	78.4 ± 67.3	78.3 ± 68.2	81.5 ± 62.8
Serum calcium (mmol/L)	2.4 ± 0.1	2.4 ± 0.1	2.4 ± 0.1
Serum zinc (μmol/L)	13.1 ± 2.5	13.5 ± 2.6	13.7 ± 3.1
Serum copper (μmol/L)	16.1 ± 3.6	16.2 ± 3.4	16.8 ± 3.8
Serum phosphorus (mmol/L)	1.2 ± 0.2	1.2 ± 0.2	1.2 ± 0.2
Education (high school or above, %)	42.3	49.8	49.0
Occupation (manual labor, %)	20.1	17.2	17.5

*Mg* magnesium, *BMI* body mass index

**Fig. 1:**
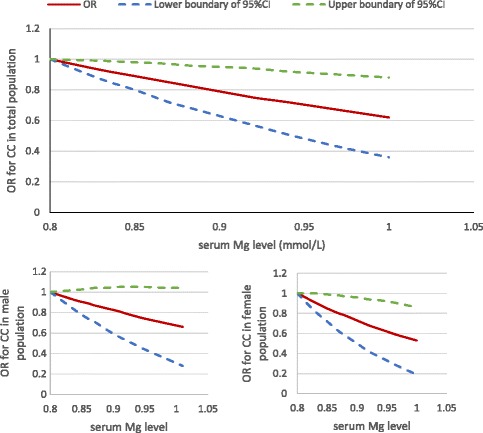
Dose-response relationship between serum magnesium (Mg) level and the odds ratio (OR) for knee chondrocalcinosis (CC) in the Xiangya Hospital Health Management Center Study. CI, confidence interval

**Fig. 2:**
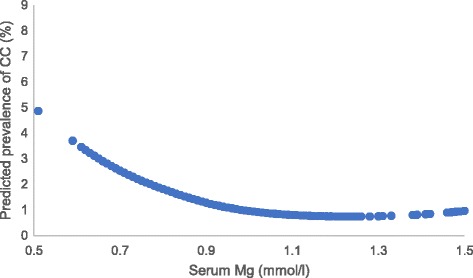
Association between serum magnesium (Mg) and predicted prevalence of chondrocalcinosis (CC) analyzed by spline regression in the Xiangya Hospital Health Management Center Study

As shown in Fig. 1, serum magnesium, even within the normal range, was inversely associated with the OR for knee chondrocalcinosis in a dose-response-relationship manner (test for trend *P* = 0.03 for total population; *P* = 0.16 for the male population; *P* = 0.05 for the female population). The prevalence of knee chondrocalcinosis (knee-specific analysis) decreased from 1.3% in the lowest tertile of serum magnesium to 0.8% in the second, and 0.6% in the highest tertile of serum magnesium. Figure 2 showed the linear association between the serum magnesium and predicted prevalence of chondrocalcinosis. After adjusting for age, sex and BMI, the ORs for knee chondrocalcinosis were 0.59 (95% CI 0.40–0.87) in the middle and 0.49 (95% CI 0.33–0.72) in the highest tertile of serum magnesium, respectively, compared with the lowest tertile (*P* value for trend <0.001). Adding each of the potential confounders (e.g., serum iron, ferritin, calcium, zinc, copper and phosphorus) into the age, sex and BMI-adjusted model, or sensitivity analysis with exclusion of participants with chronic renal failure, also did not change the results materially. The association was also consistent in men and women (Table 2). When 0.70 mmol/L was used as an additional knot for spline regression, and the category ≤0.70 was used as reference group for GEE analysis, the results did not change significantly (see Additional file 2).

**Table 2:** Association between serum Mg and knee chondrocalcinosis in the Xiangya Hospital Health Management Center Study (n = 12,631)

	Tertiles of serum Mg (mmol/L)			*P* for trend
	1 (≤0.86)	2 (0.87–0.91)	3 (≥0.92)	
Total				
*N* for knee*	9263	7438	8497	-
Knee chondrocalcinosis (%)	1.3	0.8	0.6	-
Model 1 (95% CI)	1.00 (reference)	0.59 (0.40, 0.87)	0.49 (0.33, 0.72)	<0.001
Model 2 (95% CI)	1.00 (reference)	0.59 (0.39, 0.89)	0.53 (0.36, 0.78)	0.001
Model 3 (95% CI)	1.00 (reference)	0.69 (0.36, 1.32)	0.38 (0.20, 0.71)	0.002
Model 4 (95% CI)	1.00 (reference)	0.60 (0.41, 0.89)	0.48 (0.33, 0.71)	<0.001
Model 5 (95% CI)	1.00 (reference)	0.58 (0.39, 0.88)	0.52 (0.35, 0.78)	0.001
Model 6 (95% CI)	1.00 (reference)	0.57 (0.38, 0.86)	0.49 (0.33, 0.73)	<0.001
Model 7 (95% CI)	1.00 (reference)	0.60 (0.41, 0.89)	0.48 (0.33, 0.72)	<0.001
Model 8 (95% CI)	1.00 (reference)	0.83 (0.43, 1.58)	0.46 (0.23, 0.90)	0.021
Male				
*N* for knee*	5065	4279	5058	-
Knee chondrocalcinosis (%)	1.4	0.7	0.5	-
Model 1 (95% CI)	1.00 (reference)	0.56 (0.33, 0.95)	0.43 (0.25, 0.74)	0.002
Model 2 (95% CI)	1.00 (reference)	0.54 (0.31, 0.96)	0.45 (0.26, 0.78)	0.003
Model 3 (95% CI)	1.00 (reference)	0.65 (0.27, 1.56)	0.37 (0.16, 0.84)	0.017
Model 4 (95% CI)	1.00 (reference)	0.57 (0.34, 0.98)	0.42 (0.24, 0.73)	0.002
Model 5 (95% CI)	1.00 (reference)	0.54 (0.30, 0.94)	0.45 (0.26, 0.78)	0.003
Model 6 (95% CI)	1.00 (reference)	0.54 (0.31, 0.94)	0.41 (0.24, 0.72)	0.001
Model 7 (95% CI)	1.00 (reference)	0.57 (0.33, 0.98)	0.42 (0.24, 0.73)	0.002
Model 8 (95% CI)	1.00 (reference)	0.92 (0.40, 2.11)	0.51 (0.21, 1.24)	0.134
Female				
*N* for knee*	4198	3159	3494	-
Knee chondrocalcinosis (%)	1.2	0.9	0.8	-
Model 1 (95% CI)	1.00 (reference)	0.64 (0.36, 1.13)	0.54 (0.30, 0.96)	0.034
Model 2 (95% CI)	1.00 (reference)	0.65 (0.36, 1.19)	0.60 (0.33, 1.09)	0.094
Model 3 (95% CI)	1.00 (reference)	0.78 (0.29, 2.13)	0.41 (0.14, 1.21)	0.091
Model 4 (95% CI)	1.00 (reference)	0.65 (0.37, 1.15)	0.54 (0.31, 0.96)	0.035
Model 5 (95% CI)	1.00 (reference)	0.65 (0.36, 1.18)	0.59 (0.33, 1.07)	0.081
Model 6 (95% CI)	1.00 (reference)	0.65 (0.35, 1.19)	0.60 (0.33, 1.10)	0.096
Model 7 (95% CI)	1.00 (reference)	0.65 (0.36, 1.14)	0.54 (0.30, 0.96)	0.034
Model 8 (95% CI)	1.00 (reference)	0.67 (0.24, 1.88)	0.37 (0.13, 1.12)	0.073

Model 1 included age (40–49, 50–59, 60–69, ≥70 years), body mass index (BMI) (<25, ≥25 kg/m^2^) and sex (age and BMI for the sex subgroup) (n = 12,631)

Model 2 added serum iron (tertiles) on the basis of model 1 (n = 12,149)

Model 3 added serum ferritin (tertiles) on the basis of model 1 (n = 5113)

Model 4 added serum calcium (tertiles) on the basis of model 1 (n = 12,479)

Model 5 added serum zinc (tertiles) on the basis of model 1 (n = 12,154)

Model 6 added serum copper (tertiles) on the basis of model 1 (n = 12,153)

Model 7 added serum phosphorus (tertiles) on the basis of model 1 (n = 12490)

Model 8 added education (high school or above, lower than high school) and occupation (manual labor, non-manual labor) on the basis of model 1 (n = 5844)

*Five right knees and four left knees with Kellgren-Lawrence grade 4were excluded from analysis (data from the contralateral knees were retained)

*Mg* magnesium, *N* number, *CI* confidence interval

### XO Study I

Of 1469 participants in the XO Study I, 1316 were included in the current analysis. About half (51.2%, n = 674) were women, average age was 62.9 ± 8.7 years and mean BMI was 24.2 kg/m^2^ (SD = 3.6). The prevalence of knee chondrocalcinosis was 3.1% in men and 5.0% in women (Table 3).

**Table 3:** Basic characteristics of the study population in XO Study I (n = 1316)

	Tertiles of serum Mg (mmol/L)		
	1 (≤0.89)	2 (0.90-0.95)	3 (≥0.96)
Number	490	400	426
Median Mg level (mmol/L)	0.85	0.92	1.00
Sex (female, %)	48.6	50.3	55.2
Age (years)	63.6 ± 8.9	63.3 ± 8.4	61.6 ± 8.5
50–59 (%)	37.1	36.3	46.4
60–69 (%)	36.3	40.0	34.9
≥70 (%)	26.7	23.8	18.7
BMI (kg/m^2^)	24.1 ± 3.5	24.3 ± 3.9	24.3 ± 3.4
< 25 (%)	67.6	61.0	59.9
≥25 (%)	32.4	39.0	40.1
Knee injury (yes, %)	5.5	3.8	5.2
Serum PTH (μmol/L)	52.5 ± 38.0	54.8 ± 23.1	51.3 ± 20.2
Serum iron (μmol/L)	20.2 ± 8.6	19.8 ± 7.2	20.3 ± 7.8
Serum TIBC (μmol/L)	58.3 ± 11.3	59.1 ± 11.6	60.2 ± 11.4
Serum UIBC (μmol/L)	38.2 ± 13.5	39.5 ± 13.2	40.0 ± 13.1
Serum calcium (mmol/L)	2.3 ± 0.1	2.3 ± 0.1	2.3 ± 0.1
Serum 25(OH)D (μg/L)	25.8 ± 11.3	24.9 ± 9.9	25.9 ± 10.9
Serum zinc (μmol/L)	17.3 ± 3.5	18.5 ± 3.7	19.9 ± 6.1
Serum copper (μmol/L)	16.3 ± 3.2	16.4 ± 3.7	16.8 ± 3.5
Serum phosphorus (mmol/L)	1.2 ± 0.2	1.2 ± 0.2	1.2 ± 0.2
Education (educated, %)	70.2	66.5	67.8
Occupation (farmer, %)	85.5	87.3	85.7

*Mg* magnesium, *BMI* body mass index, *PTH* parathyroid hormone, *TIBC*, total iron binding capacity, *UIBC* unsaturated iron-binding capacity

**Fig. 3:**
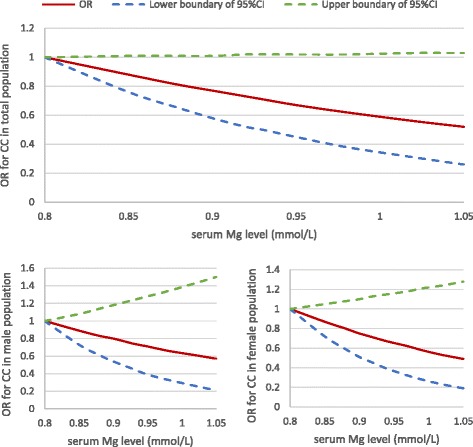
Dose-response relationship between serum magnesium (Mg) and the odds ratio (OR) for knee chondrocalcinosis (CC) in the XO Study I. CI, confidence interval

**Fig. 4:**
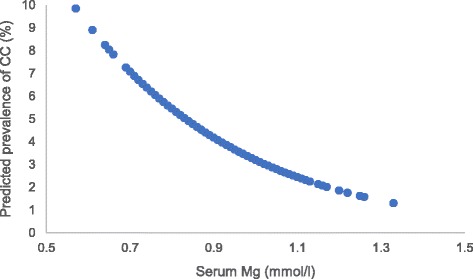
Association between serum magnesium (Mg) and predicted prevalence of chondrocalcinosis (CC) analyzed by spline regression in the XO Study I

Serum magnesium, even within the normal range, was inversely associated with the OR for prevalence of knee chondrocalcinosis in a dose-response-relationship manner (Fig. 3, test for trend *P* = 0.06 for the total population; *P* = 0.25 for the male population; *P* = 0.14 for the female population). As shown in Table 4, the prevalence of knee chondrocalcinosis (knee-specific analysis) decreased from 4.8% in the lowest tertile of serum magnesium to 3.2% in the second, and 2.0% in the highest tertile of serum magnesium. Figure 4 shows the linear association between the serum magnesium and predicted prevalence of chondrocalcinosis. After adjusting for age, sex and BMI, compared with the lowest tertile, ORs for knee chondrocalcinosis were 0.67 (95% CI 0.34–1.30) in the middle and 0.45 (95% CI 0.21–0.94) in the highest tertile of serum magnesium, respectively (*P* value for trend = 0.030). Adding each of the potential confounders (e.g., knee injury, serum parathyroid hormone, iron, total iron binding capacity, unsaturated iron binding capacity, calcium, 25(OH)D, zinc, copper and phosphorus) into the age, sex and BMI-adjusted model, or sensitivity analysis with exclusion of participants with chronic renal failure or who used diuretics, also did not change the results materially. The association was consistent in men and women (Table 4). When 0.70 mmol/L was used as an additional knot for spline regression, and the category ≤0.70 was used as the reference group for GEE analysis, the results did no change significantly (see Additional file 2).

**Table 4:** Association between serum Mg and knee chondrocalcinosis of study population in the XO Study I (n = 1316)

	Tertiles of serum Mg (mmol/L)			*P* for trend
	1 (≤0.89)	2 (0.90–0.95)	3 (≥0.96)	
Total				
*N* for knee*	977	793	848	-
Knee chondrocalcinosis (%)	4.8	3.2	2.0	-
Model 1 (95% CI)	1.00 (reference)	0.67 (0.34, 1.30)	0.45 (0.21, 0.94)	0.030
Model 2 (95% CI)	1.00 (reference)	0.68 (0.35, 1.32)	0.45 (0.22, 0.94)	0.046
Model 3 (95% CI)	1.00 (reference)	0.73 (0.37, 1.47)	0.41 (0.18, 0.93)	0.028
Model 4 (95% CI)	1.00 (reference)	0.71 (0.35, 1.41)	0.41 (0.18, 0.91)	0.025
Model 5 (95% CI)	1.00 (reference)	0.73 (0.37, 1.46)	0.42 (0.19, 0.94)	0.032
Model 6 (95% CI)	1.00 (reference)	0.71 (0.36, 1.41)	0.40 (0.18, 0.91)	0.024
Model 7 (95% CI)	1.00 (reference)	0.67 (0.34, 1.30)	0.45 (0.21, 0.94)	0.030
Model 8 (95% CI)	1.00 (reference)	0.69 (0.35, 1.34)	0.45 (0.21, 0.95)	0.032
Model 9 (95% CI)	1.00 (reference)	0.71 (0.36, 1.42)	0.43 (0.20, 0.95)	0.020
Model 10 (95% CI)	1.00 (reference)	0.68 (0.35, 1.33)	0.41 (0.19, 0.88)	0.034
Model 11 (95% CI)	1.00 (reference)	0.68 (0.35, 1.32)	0.45 (0.21, 0.95)	0.033
Model 12 (95% CI)	1.00 (reference)	0.65 (0.33, 1.28)	0.46 (0.21, 0.96)	0.036
Male				
*N* for knee*	502	397	380	-
Knee chondrocalcinosis (%)	3.8	2.5	1.1	-
Model 1 (95% CI)	1.00 (reference)	0.72 (0.26, 2.01)	0.34 (0.08, 1.37)	0.115
Model 2 (95% CI)	1.00 (reference)	0.75 (0.28, 2.05)	0.34 (0.08, 1.38)	0.114
Model 3 (95% CI)	1.00 (reference)	0.81 (0.29, 2.30)	0.39 (0.09, 1.66)	0.195
Model 4 (95% CI)	1.00 (reference)	0.78 (0.28, 2.21)	0.39 (0.09, 1.62)	0.184
Model 5 (95% CI)	1.00 (reference)	0.87 (0.31, 2.48)	0.40 (0.10, 1.64)	0.198
Model 6 (95% CI)	1.00 (reference)	0.84 (0.30, 2.35)	0.39 (0.09, 1.60)	0.183
Model 7 (95% CI)	1.00 (reference)	0.72 (0.26, 2.00)	0.34 (0.09, 1.37)	0.117
Model 8 (95% CI)	1.00 (reference)	0.65 (0.19, 2.25)	0.47 (0.11, 1.88)	0.254
Model 9 (95% CI)	1.00 (reference)	0.81 (0.27, 2.42)	0.21 (0.04, 1.08)	0.077
Model 10 (95% CI)	1.00 (reference)	0.72 (0.26, 2.00)	0.17 (0.04, 0.83)	0.024
Model 11 (95% CI)	1.00 (reference)	0.63 (0.23, 1.74)	0.32 (0.08, 1.32)	0.094
Model 12 (95% CI)	1.00 (reference)	0.67 (0.24, 1.93)	0.34 (0.08, 1.38)	0.116
Female				
*N* for knee*	475	396	468	-
Knee chondrocalcinosis (%)	5.9	3.8	2.8	-
Model 1 (95% CI)	1.00 (reference)	0.64 (0.27, 1.52)	0.50 (0.20, 1.23)	0.128
Model 2 (95% CI)	1.00 (reference)	0.65 (0.27, 1.55)	0.50 (0.20, 1.24)	0.130
Model 3 (95% CI)	1.00 (reference)	0.66 (0.27, 1.65)	0.40 (0.15, 1.10)	0.069
Model 4 (95% CI)	1.00 (reference)	0.62 (0.25, 1.54)	0.40 (0.14, 1.09)	0.067
Model 5 (95% CI)	1.00 (reference)	0.66 (0.26, 1.63)	0.42 (0.15, 1.17)	0.090
Model 6 (95% CI)	1.00 (reference)	0.63 (0.25, 1.58)	0.41 (0.15, 1.10)	0.071
Model 7 (95% CI)	1.00 (reference)	0.64 (0.27, 1.53)	0.50 (0.20, 1.23)	0.126
Model 8 (95% CI)	1.00 (reference)	0.66 (0.27, 1.59)	0.50 (0.20, 1.23)	0.129
Model 9 (95% CI)	1.00 (reference)	0.66 (0.27, 1.59)	0.53 (0.21, 1.33)	0.174
Model 10 (95% CI)	1.00 (reference)	0.66 (0.27, 1.60)	0.53 (0.21, 1.32)	0.171
Model 11 (95% CI)	1.00 (reference)	0.68 (0.29, 1.64)	0.52 (0.21, 1.28)	0.149
Model 12 (95% CI)	1.00 (reference)	0.63 (0.26, 1.50)	0.48 (0.19, 1.21)	0.116

Model 1 included age (50–59, 60–69, ≥70 years), body mass index (BMI) (<25, ≥25 kg/m^2^) and sex (age and BMI for the sex subgroup) (n = 1316)

Model 2 added knee injury on the basis of model 1 (n = 1316)

Model 3 added serum parathyroid hormone (tertiles) on the basis of model 1 (n = 1250)

Model 4 added serum iron (tertiles) on the basis of model 1 (n = 1250)

Model 5 added serum total iron binding capacity (tertiles) on the basis of model 1 (n = 1241)

Model 6 added serum unsaturated iron binding capacity (tertiles) on the basis of model 1 (n = 1241)

Model 7 added serum calcium (tertiles) on the basis of model 1 (n = 1316)

Model 8 added serum 25(OH)D (tertiles) on the basis of model 1 (n = 1306)

Model 9 added serum zinc (tertiles) on the basis of model 1 (n = 1292)

Model 10 added serum copper (tertiles) on the basis of model 1 (n = 1292)

Model 11 added serum phosphorus (tertiles) on the basis of model 1 (n = 1316)

Model 12 added education (educated, non-educated) and occupation (farmer, non-farmer) on the basis of model 1 (n = 1316)

*Six right knees and eight left knees with Kellgren-Lawrence grade 4 were excluded from analysis (data from the contralateral knees were retained)

*Mg* magnesium, *CI* confidence interval

## Discussion

In two population-based cross-sectional studies, we found that serum magnesium, even within the normal range, was inversely associated with the prevalence of knee chondrocalcinosis in a dose-response-relationship manner. In both studies, prevalence of knee chondrocalcinosis among subjects in the highest tertile of serum magnesium was approximately 50% lower than those in the lowest tertile of serum magnesium. Such an association was consistent in men and women.

A few case-reports and case-series studies have shown that chondrocalcinosis often co-occurs with hypomagnesemia or with hypomagnesemia owing to bowel syndrome and Gitelmen syndrome [7–11]. For example, in 2007 Richette et al. compared serum magnesium and the prevalence of chondrocalcinosis in 72 patients with intestinal failure and 72 age-matched and sex-matched patients with back pain [6]. They found that serum magnesium was significantly lower (*P* < 0.001) among patients with intestinal failure (0.75 mmol/L) than those with back pain (0.81 mmol/L). On the other hand, the prevalence of chondrocalcinosis was sevenfold higher (*P* = 0.006) among patients with intestinal failure (16.6%) than among patients with back pain (2.7%). Furthermore, serum magnesium, globular magnesium and 24-hour urinary magnesium were significantly lower in patients with intestinal failure than those with back pain.

Several in vivo studies have demonstrated that decreasing magnesium intake induces calcification formation in different animal models [21–24]. However, few in vivo studies, if any, have examined the effect of magnesium supplementation or intake on the inhibition of cartilage calcification. In a double-blind randomized clinical trial assessing the effect of a magnesium carbonate supplement among patients with chronic pyrophosphate arthropathy, there was no significant difference in the radiographic appearance of chondrocalcinosis between the treatment and placebo groups; however, the treatment group had a uniform trend towards improvement in pain, stiffness, effusion, tenderness and overall subjective and objective assessment compared with the placebo group over a 6-month period [25]. This finding could be explained by the physiologic N-methyl-D-aspartate (NMDA) receptor antagonist property of magnesium, and consequently, increasing serum magnesium level may reduce the incidence of symptomatic calcium-deposition-related diseases [26]. Furthermore, in a large population-based study, Zhang et al. reported that the prevalence of knee chondrocalcinosis was much lower among participants in the Beijing Osteoarthritis Study than in their counterparts in the Framingham Study (age-standardized prevalence ratios = 0.34 in men and 0.43 in women); however, no difference in the levels of magnesium in the tap drinking water was found between the two cities [5].

Several mechanisms linking magnesium and chondrocalcinosis have been postulated [27]. These include that high serum magnesium may inhibit the formation of calcium phosphate apatite and of calcium-acidic phospholipid-phosphate complexes [28, 29], may increase the expression of calcification inhibitors (e.g., matrix gla protein, osteopontin, and bone morphogenetic protein 7), may decrease the expression of calcification promoters (e.g., alkaline phosphatase, bone morphogenetic protein 2 and runt-related transcription factor 2) and apoptosis [30–33], may activate calcium-sensing receptors [34–36], modulate vitamin D receptor, fibroblast growth factor-1 receptor and its co-receptor klotho [36], or may block various calcium channels to impair excessive calcium uptake [37, 38].

Several characteristics of our study are noteworthy. First, compared with other studies, the sample size of the current two studies was relatively large, and the prevalence of knee chondrocalcinosis found in these two studies (1.4% in the Xiangya Hospital Health Management Center Study and 4.1% in the XO Study I) was similar to another population-based study (i.e., the Beijing Osteoarthritis Study) conducted among residents in Beijing (1.8% in men and 2.7% in women) [5]. These findings suggest that the prevalence of knee chondracalcinosis is lower than in the predominantly white participants in the Framingham Study [5]. In addition, we also showed that prevalence of hypomagnesemia appeared to be lower among the participants in the current two studies than that among subjects in the Rotterdam Study [39]. Second, an inverse association between serum magnesium levels and prevalence of knee chondrocalcinosis was found in our two study populations and across the sex categories, and the magnitude of association was also quite similar when several potential confounders (e.g., knee injury, serum parathyroid hormone, iron, ferritin, total iron binding capacity, unsaturated iron binding capacity, calcium, copper, zinc, phosphorus and 25(OH)D) was added into the age, sex and BMI adjusted model, respectively, indicating the robustness of our findings. Third, we also showed that even within the normal range of serum magnesium, the levels of magnesium were still inversely associated with the prevalence of knee chondrocalcinosis in a dose-response-relationship manner. This finding suggests that increased serum magnesium not only among subjects with hypomagnesium but also among subjects who were within the normal range of magnesium may help to reduce the risk of chondrocalcinosis.

The prevalence of radiographic chondrocalcinosis in the XO Study I was two to three times higher than that in the Xiangya Hospital Health Management Center Study. Such a difference may be due to the age difference between the two study populations in that the average age in the XO Study I (62.9 ± 8.7 years) was almost 10 years older than that in the Xiangya Hospital Health Management Center Study (52.3 ± 8.0 years). While the age distribution is similar in the XO Study I, and the Beijing Osteoarthritis Study restricted participants to 60 years and older, the prevalence in the XO Study I was higher than in the Beijing Osteoarthritis study. This may because of the heavy physical labor (82.4% of men and 91.6% of women were farmers) and frequent climbing (mountain area) of participants in the XO Study I, which of course warrants further study for confirmation.

Our study has some limitations. First, this is a cross-sectional study, thus, we cannot be certain that the causal relationship between serum levels of magnesium and prevalence of knee chondrocalcinosis. Second, we did not assess the dietary intake of magnesium in relation to the prevalence of knee chondrocalcinosis in the present study. While previous studies have shown that dietary intake of magnesium is strongly associated with the serum levels of magnesium [40, 41], studies of dietary intake of magnesium and risk of knee chondrocalcinosis are warranted. Third, we did not use other views of the knee (e.g., lateral or skyline) to ascertain radiographic choncrocalcinosis; thus, it may have resulted in underestimation of the prevalence of radiographic CPPD. However, we postulate that such misclassification of the prevalence of knee CPPD is likely to be non-differential; thus the association between serum magnesium levels and prevalence of knee radiographic CPPD may be even stronger than we have observed. In addition, as almost half of cases of wrist, hip, symphysis pubis, and metacarpophalangeal joint chondrocalcinosis occur without knee chondrocalcinosis [42], the examination of the association of magnesium levels with a predisposition to form CPPD crystals would ideally include radiographs from other joint sites. Fourth, a synovial fluid test taken from the joint should be more sensitive and specific to detect crystals than radiographs, and some of the articular CPPD deposition (alone or mixed with BCP [2, 3]) may be too small to be detected on radiographs; thus, our estimate of the prevalence of knee chondrocalcinosis is likely to be underestimated. However, as we stated previously, that prevalence estimate of knee chondrocalcinosis was similar to another study [5] conducted in Chinese subjects that used the same protocol to assess the presence of knee chondrocalcinosis. The proportion of persons with chondrocalcinosis identified on radiographs who develop clinical symptoms related to this is small. Fifth, data on some other potential confounders (e.g., osteoporosis and use of bisphosphonates) were not collected in either study and could impact the results. We performed sensitivity analysis to assess to what extent the residual confounding could explain the association, using the E-value proposed by VanderWeele and Ding [43]. The observed OR of 0.49 for prevalence with the highest levels of magnesium in Xiangya Hospital Health Management Center Study could be explained if an OR of an unmeasured confounder with either high serum magnesium or with the prevalence of chondrocalcinosis is at least 3.50, which is above and beyond the measured confounders. There should be a similar magnitude of residual confounders to explain the findings in the XO Study I. To our best knowledge we are unaware that there is such a strong confounder between magnesium and the prevalence of chondrocalcinosis.

Our study findings have potential clinical implications. The existing in vivo and in vitro studies showed that magnesium supplementation may have a protective effect on cartilage or chondrocytes [44]. Two large epidemiological studies conducted in twins and in the general population reported an inverse association between serum magnesium levels and radiographic knee osteoarthritis [12, 45]. Furthermore, several studies have reported that chondrocalcinosis may be a potential risk factor for osteoarthritis [46–52]. Thus, it is not unreasonable to speculate that increasing serum magnesium may be beneficial in decreasing the risk of osteoarthritis through reducing the incidence of chondrocalcinosis. Future studies are warranted to test this hypothesis.

## Conclusions

We found that low serum magnesium, even within the normal range, was associated with higher prevalence of knee chondrocalcinosis in a dose-response-relationship manner. Future studies of dietary intake, including magnesium supplementary intake, on the risk of chondrocalcinosis are warranted.

## Additional files

Additional file 1: Measuring methods and reliability data of potential confounders. (DOCX 15 kb)

Additional file 2: Table S1. Association between serum Mg and knee chondrocalcinosis in the Xiangya Hospital Health Management Center Study (n = 12,631). **Table S2.** Association between serum Mg and knee chondrocalcinosis in the XO Study I (n = 1316). **Figure S1.** Association between serum Mg and OR of CC analyzed by spline regression (3 knots, 0,7 as reference) in the Xiangya Hospital Health Management Center Study (*P* = 0.049). Mg, magnesium; CC, chondrocalcinosis; OR, odds ratio; CI, confidence interval. **Figure S2.** Association between serum Mg and OR of CC analyzed by spline regression (3 knots, 0,7 as reference) in the XO Study I (*P* = 0.060). Mg, magnesium; CC, chondrocalcinosis; OR, odds ratio; CI, confidence interval. (DOCX 1720 kb)

## Abbreviations

BCP: Basic calcium phosphate
BMI: Body mass index
CPPD: Calcium pyrophosphate deposition
K-L: Kellgren-Lawrence
NMDA: N-methyl-D-aspartate
OAI: Osteoarthritis Initiative
ORs: Odds ratios
XO Study I: Xiangya Osteoarthritis Study I
