# Supplementary material for: Dose-response relationship between lower serum magnesium level and higher prevalence of knee chondrocalcinosis
Source: Arthritis Res Ther. 2017 Oct 24;19:236. doi: 10.1186/s13075-017-1450-6 (PMC5655810; doi:10.1186/s13075-017-1450-6)
Supplement: Supplementary file 1 — Measuring methods and reliability data of potential confounders. (DOCX 15 kb) [file 13075_2017_1450_MOESM1_ESM.docx]

**Measuring methods and reliability data of potential confounders**

**Serum parathyroid hormone**

The serum parathyroid hormone concentration were measured using the electrochemiluminescence method by Roche Cobas E601. The intra-assay coefficients of variation were 2.0% (21.9 pg/ml), 1.2% (35 pg/ml) and 1.1% (123 pg/ml) for serum parathyroid hormone, and the inter-assay coefficients of variation were 3.4% (23.2 pg/ml), 2.5% (80.9 pg/ml) and 2.8% (240 pg/ml) for serum parathyroid hormone.

**Serum iron**

The serum iron concentration was measured using the Ferene method by Roche modular P800. The inter-assay coefficients of variation were 1.426% (11.6 umol/L) and 0.6% (44.5 umol/L) for serum iron. The intra-assay coefficients of variation were 1.55% (43.95 umol/L) and 1.03% (11.29 umol/L) for serum iron.

**Serum ferritin**

The serum ferritin concentration was measured using the chemiluminescence method by Beckman Coulter DXI800. The inter-assay coefficients of variation were 4.1% (37.2 ng/ml), 4.3% (118.9 ng/ml) and 6.3% (311.8 ng/ml) for serum ferritin. The intra-assay coefficients of variation were 2.6% (37.2 ng/ml), 3.6% (118.9 ng/ml) and 3.9% (311.8 ng/ml) for serum ferritin.

**Serum total iron binding capacity**

The serum iron concentration and unsaturated iron-bonding capacity were measured using the colorimetric assay by Roche Cobas C501 for calculating the serum total iron binding capacity. The intra-assay coefficients of variation were 0.3% (538 ug/dl) and 2.8% (92.2 ug/dl) for unsaturated iron-bonding capacity, and the inter-assay coefficients of variation were 4.7% (92.2 ug/dl), 3.1% (136 ug/dl) for unsaturated iron-bonding capacity.

**Serum calcium**

The serum calcium concentration was measured using the Arsenazo III method by Beckman Coulter AU 5800. The inter-assay coefficients of variation were 1.03% (3.01 mmol/L) and 0.86% (2.33 mmol/L) for serum calcium. The intra-assay coefficients of variation were 0.86% (2.35 mmol/L) and 0.58% (3.56 mmol/L) for serum calcium.

**Serum copper**

The serum copper concentration was measured using the PAESA method by Roche modular P800. The inter-assay coefficients of variation were 6.15% (9.8 umol/L) and 4.33% (16.5 umol/L) for serum copper. The intra-assay coefficients of variation were 5.17% (16.72 umol/L) and 5.37% (8.86 umol/L) for serum copper.

**Serum zinc**

The serum zinc concentration was measured using the PAPS method by Roche modular P800. The inter-assay coefficients of variation were 4.44% (10.1 umol/L) and 2.50% (18.5 umol/L) for serum zinc. The intra-assay coefficients of variation were 2.86% (18.74 umol/L) and 3.42% (9.49 umol/L) for serum zinc.

**Serum phosphorus**

The serum phosphate concentration was measured using the phosphomolybdate method by Beckman Coulter AU 5800. The inter-assay coefficients of variation were 2.38% (2.48 mmol/L) and 2.41% (1.16 mmol/L) for serum phosphate. The intra-assay coefficients of variation were 0.66% (1.12 mmol/L) and 0.33% (2.84 mmol/L) for serum phosphate.

**Serum vitamin D**

The serum vitamin D concentration were measured using the electrochemiluminescence method by Roche Cobas E601. The intra-assay coefficients of variation were 6.8% (8.35 ng/ml), 5.2% (15.8 ng/ml), 3.9% (28.3 ng/ml) and 2.2% (69.6 ng/ml) for serum vitamin D, and the inter-assay coefficients of variation were 13.1% (8.35 ng/ml), 7.5% (15.8 ng/ml), 6.5% (28.3 ng/ml) and 3.4% (69.6 ng/ml) for serum vitamin D.
