# Supplementary material for: Dose-response relationship between lower serum magnesium level and higher prevalence of knee chondrocalcinosis
Source: Arthritis Res Ther. 2017 Oct 24;19:236. doi: 10.1186/s13075-017-1450-6 (PMC5655810; doi:10.1186/s13075-017-1450-6)
Supplement: Supplementary file 2 — Association between serum Mg and knee chondrocalcinosis in the Xiangya Hospital Health Management Center Study (n = 12,631). Table S2. Association between serum Mg and knee chondrocalcinosis in the XO Study I (n = 1316). Figure S1. Association between serum Mg and OR of CC analyzed by spline regression (3 knots, 0,7 as reference) in the Xiangya Hospital Health Management Center Study (P = 0.049). Mg, magnesium; CC, chondrocalcinosis; OR, odds ratio; CI, confidence interval. Figure S2. Association between serum Mg and OR of CC analyzed by spline regression (3 knots, 0,7 as reference) in the XO Study I (P = 0.060). Mg, magnesium; CC, chondrocalcinosis; OR, odds ratio; CI, confidence interval. (DOCX 1720 kb) [file 13075_2017_1450_MOESM2_ESM.docx]

Table S1 Association between serum Mg and knee chondrocalcinosis in Xiangya Hospital Health Management Center Study (n=12631)

|  | Quartiles of serum Mg (mmol/L) | | | | P for trend |
| --- | --- | --- | --- | --- | --- |
|  | ≤0.70 | 0.71-0.86 | 0.87-0.91 | ≥0.92 |  |
| Total |  |  |  |  |  |
| N for knee* | 135 | 9128 | 7438 | 8552 | - |
| Knee CHONDROCALCINOSIS (%) | 3.0 | 1.3 | 0.8 | 0.6 | - |
| Model 1 (95% CI) | 1.00 (reference) | 0.56  (0.13, 2.52) | 0.34  (0.07, 1.54) | 0.28  (0.06, 1.26) | <0.001 |

Model 1 included age, BMI and sex (n=12631).

*Five right knees and four left knees with K-L 4 grade were excluded for analysis (data from the contralateral knees were retained).

Mg, magnesium; CHONDROCALCINOSIS, chondrocalcinosis; N, number; BMI, body mass index; OR, odds ratio; CI, confidence interval.

Table S2 Association between serum Mg and knee chondrocalcinosis of XO Study I (n=1316)

|  | Quartiles of serum Mg (mmol/L) | | | | P for trend |
| --- | --- | --- | --- | --- | --- |
|  | ≤0.70 | 0.71-0.89 | 0.90-0.95 | ≥0.96 |  |
| Total |  |  |  |  |  |
| N for knee* | 34 | 943 | 793 | 848 | - |
| Knee CHONDROCALCINOSIS (%) | 14.7 | 4.5 | 3.2 | 2.0 | - |
| Model 1 (95% CI) | 1.00 (reference) | 0.31  (0.07, 1.40) | 0.23  (0.05, 1.05) | 0.15  (0.03, 0.73) | 0.009 |

Model 1 included age, BMI and sex (n=1316).

*Six right knees and eight left knees with K-L 4 grade were excluded for analysis (data from the contralateral knees was retained).

Mg, magnesium; CHONDROCALCINOSIS, chondrocalcinosis; N, number; BMI, body mass index; OR, odds ratio; CI, confidence interval.

Figure S1 Association between serum Mg and OR of CC by spline regression (3 knots, 0,7 as reference) in Xiangya Hospital Health Management Center Study (P=0.049). Mg, magnesium; CC, chondrocalcinosis; OR, odds ratio; CI, confidence interval.

Figure S2 Association between serum Mg and OR of CC by spline regression (3 knots, 0,7 as reference) in XO Study I (P=0.060). Mg, magnesium; CC, chondrocalcinosis; OR, odds ratio; CI, confidence interval.
